# Severity of Coronary Artery Disease by Friesinger Score Among Women With Acute Coronary Syndrome in a Tertiary Care Hospital in Bangladesh

**DOI:** 10.7759/cureus.54617

**Published:** 2024-02-21

**Authors:** Ayesha Siddika, Fazila-Tun-Nesa Malik, Md Kalimuddin, Nahidul Hasan, Nazir Ahmed, Mohammad Badiuzzaman, Dhiman Banik, Tawfiq S Huq, Mir Ishraquzzaman, Faria Rahman

**Affiliations:** 1 Internal Medicine, Bangladesh College of Physicians and Surgeons, Dhaka, BGD; 2 Cardiology, National Heart Foundation Hospital & Research Institute, Dhaka, BGD; 3 Interventional Cardiology, National Heart Foundation Hospital & Research Institute, Dhaka, BGD

**Keywords:** coronary artery disease, premenopausal and postmenopausal women, friesinger score, coronary angiographic severity, acute coronary syndrome (acs)

## Abstract

Background: The chance of coronary artery disease (CAD) is much higher in women who have gone through menopause than in those who have not, owing to hormonal defense against atherosclerosis. More advanced CAD and several comorbidities were observed in postmenopausal women. Nevertheless, there is a paucity of information comparing the angiographic severity of acute coronary syndrome (ACS) in premenopausal and postmenopausal women of different ages. This research sought to determine the Friesinger score's use in evaluating the degree of CAD in premenopausal and postmenopausal women with ACS.

Methods: A total of 145 female patients with ACS were included in this cross-sectional observational research. Depending on the stage of menopause, they were categorized into two groups: group I (premenopausal) and group II (postmenopausal). The study examined the differences in clinical data and the severity of coronary angiographic features based on the Friesinger score between the premenopausal and postmenopausal ACS groups.

Results: A statistically significant difference (p = 0.001) was found in the mean age of premenopausal ACS patients, which was 41.53 ± 5.45 years, and postmenopausal ACS patients, which was 57.23 ± 7.45 years. Between the premenopausal group (31.4% vs. 17.1%; p = 0.04 and 31.4% vs. 15.7%; p = 0.002) and postmenopausal group (48.6% vs. 20%; p = 0.001), there was a greater prevalence of normal coronary angiography, single-vessel disease, and triple-vessel disease. Comparing the postmenopausal group to the premenopausal group, the high to intermediate Friesinger score (11-15) was found to be considerably higher (2.9% vs. 1.4%; 72.9% vs. 50%; p = 0.003).

Conclusion: Prior to menopause, single-vessel disease and normal coronary angiography were more common, whereas postmenopausal individuals had triple-vessel disease. The postmenopausal group's CAD was found to be more severe than the premenopausal group's according to the Friesinger score used for severity evaluation.

## Introduction

Women tend to develop heart disease much later than males do, and young women are less likely to develop heart disease because estrogen has a vascular protective effect that helps prevent atherosclerosis [1]. The truth is that irrespective of race or ethnicity, coronary artery disease (CAD) is a significant cause of death in women [2,3].

Every woman experiences menopause, a natural biological occurrence that is indicated by the end of her menstrual cycle, in her late 40s or early 50s. Women's estrogen levels during menopause are around one-third of what they were before menopause. After menopause, there are changes in the synthesis of female hormones that lead to an increased risk of ischemic heart disease and cerebrovascular accident (CVA), which are the leading causes of morbidity and mortality among women in industrialized and developing nations alike [4,5].

The premenopausal women had a higher prevalence of angiographically normal epicardial coronaries, which suggests nonatherosclerotic disease. The coronary angiographic profile showed a clear difference in the pattern of CAD between the postmenopausal and premenopausal women. Premenopausal women were more likely to have single-vessel disease even in those with large coronary lesions, but multi-vessel disease was more prevalent in postmenopausal women, with triple-vessel disease accounting for the majority of cases [6]. For premenopausal women, single-vessel involvement was substantially greater, whereas for postmenopausal women, triple-vessel involvement was significantly higher. Postmenopausal women's Friesinger severity evaluation scores were also noticeably higher [5].

However, there have not been enough studies done on premenopausal women because of the low incidence of CAD in this group [7]. This study's goal was to use the Friesinger score to compare premenopausal and postmenopausal women with acute coronary syndrome (ACS) in terms of the severity of their CAD.

## Materials and methods

A medical teaching hospital and research facility in central Bangladesh conducted this observational cross-sectional investigation. Subjects in the study were enrolled only after receiving written informed consent from each participant and institutional ethical clearance from the National Heart Foundation Hospital & Research Institute's Post Graduate Research Committee (reference number: N.H.F.H.& R.I 4-14/7/Ad/04; dated: December 28, 2021). This study included women with ACS who were premenopausal or postmenopausal and were admitted to the Cardiology Department of the National Heart Foundation Hospital & Research Institute in Dhaka, Bangladesh, between December 28, 2021 and November 28, 2022 (a period of one year). This study excluded participants with congenital heart disorders, associated valvular heart diseases, cardiomyopathy, severely severe concurrent conditions (severe dementia, advanced cancer), and unwillingness to participate.

One definition of postmenopause was the absence of menstrual blood for a full year or a history of hysterectomy [8]. Premenopause women included women who had not gone through menopause or undergone an oophorectomy [9].

By using an ECG and cardiac biomarker, the patients' diagnosis of ACS was confirmed, and it was consistent with their clinical presentation. In this investigation, the Friesinger score and the whole coronary angiographic profile were noted. There are 15 possible scores on the Friesinger index, ranging from 0. The left anterior descending artery (LAD), left circumflex artery (LCX), and right coronary artery (RCA) were the three major coronary arteries that were evaluated independently from 0 to 5 [10,11]: score 0: no disease; score 1: lesion <50% area stenosis; score 2: single lesion >50% but <90%; score 3: multiple lesions >50% but <90%; score 4: 90% lesion area; score 5: total obstruction with 100% lesion area.

Statistical analysis

G*Power version 3.1.9.4 was used to calculate the sample size using the mean difference between two independent means (two groups), effect size of 0.50, alpha error of 0.05, power of 80%, and location ratio of 1. There were 140 samples in all. Using SPSS version 22 software (IBM Corp., Armonk, NY), data input was completed. Version 22 of the SPSS software was used for all statistical analyses. For the categorical data, percentage and frequency were given, while mean ± SD was used for the continuous variables. With the Student's t-test, the means of continuous variables were compared. Correlation analyses were done by Pearson correlation coefficient for continuous variables. The significance of the result as determined by a 95% confidence interval and a p-value <0.05 was considered to be statistically significant.

## Results

This study comprised 140 premenopausal and postmenopausal women diagnosed with ACS who had coronary angiography. Patients were divided into two groups based on whether or not they were menopausal. Premenopausal women with ACS made up group I and postmenopausal women with ACS made up group II. The group I patients' mean age was 41.56 ± 5.46 years, whereas the group II patients' mean age was 57.23 ± 7.45 years, as shown in Table 1.

**Table 1:** Age distribution of the patients between the groups

Age (year)	Group I	Group II	p-value
≤40	28 (40.0)	0 (0.0)	
41-50	41 (58.6)	13 (18.6)	
51-60	1 (1.4)	41 (58.6)	
>60	0 (0.0)	16 (22.9)	
Total	70 (100.0)	70 (100.0)	
Mean ± SD	41.56 ± 5.46	57.23 ± 7.46	<0.001

Among the respondents (Table 2), a normal Friesinger score (0) was found in 24 (34.3%) patients in group I and seven (10%) patients in group II. Low Friesinger score (1-4) was found in nine (12.8%) and six (8.6%) patients in group I and group II, respectively. Hence, normal and low Friesinger scores were higher in group I but normal Friesinger scores were statistically significant in group I (p = 0.001). Intermediate Friesinger scores (5-10) were found in 38 (54.2%) patients in group I and in 56 (80.0%) patients in group II, which was statistically significant (p = 0.003). A high Friesinger score (11-15) was found in one patient (1.4%) in group I and two patients (2.9%) in group II, respectively, but it was not statistically significant (p = 0.378).

**Table 2:** Comparison of Friesinger scores between the groups (n = 140)

Friesinger score	Group I (n = 70)	Group II (n = 70)	p-value
Normal (0)	24 (34.3)	7 (10)	0.001
Low (1-4)	9 (12.8)	6 (8.6)	0.234
Intermediate (5-10)	38 (54.2)	56 (80.0)	0.003
High (11-15)	1 (1.4)	2 (2.9)	0.378
Total	70 (100.0)	70 (100.0)	

## Discussion

The mean age of group I patients was 41.53 ± 5.45 years, whereas the mean age of group II patients was 57.23 ± 7.45 years in this study. Ages 41 to 45 years made up the majority of group I patients. The study conducted by Ahmed et al. [5] revealed that group II patients had a mean age of 41.6 ± 3.8 as opposed to 56.0 ± 7.2. This finding was consistent with the majority of patients in the group being between 56 and 60 years old and also matched with Siddika et al.'s study [12].

Significantly more normal coronary angiography and single-vessel disease are seen in premenopausal women. The increased frequency of microvascular dysfunction in premenopausal women might be the cause of this normal coronary angiography. Because of diabetes mellitus, older age, and higher acute myocardial infarction, triple-vessel disease was considerably more common in postmenopausal women, which was in line with a study by Ahmed et al. [5]. Additionally, they found that premenopausal women had a greater incidence of single-vessel disease (24% vs. 52%), whereas postmenopausal women had a higher prevalence of triple-vessel disease (12% vs. 40%, p < 0.05). These results agreed with what we had found.

The left anterior descending artery, right coronary artery, and left circumflex artery were the most often involved coronary artery lesions in groups I and II, according to the distribution of lesions. Compared to group II, group I had a higher prevalence of left main coronary artery involvement.

Between the two groups, the involvement of the left anterior descending artery and right coronary artery is statistically significant (p < 0.001).

The Friesinger score was used to analyze the severity of the study patients. Normal score (0) was found in group I patients to be 34.3% vs. 10%, and intermediate score (5-10) was found in group II patients to be 80.0% vs. 54.2%. This difference is highly statistically significant (p < 0.005). Ahmed et al. analyzed 100 pre- and postmenopausal women with ACS and showed that low Friesinger scores were more common in premenopausal women (40% vs. 16%) and higher scores were more common in postmenopausal women (6% vs. 32%) [5]. These results matched our study findings.

Lesions in group I were primarily limited to single or double blood vessels, as shown by the much lower Friesinger scores of the patients. The likelihood of inflammation, coronary spasm, plaque erosion, and rupture was presumably higher in young women. However, because they had a longer illness duration and a longer disease course, postmenopausal women had more complicated clinical symptoms and easier collateral circulation formation.

Limitations of the study

Since the study was conducted at a Dhaka public hospital and the participants were hand-picked, it is not possible to draw conclusions about how representative this sample is of Bangladesh's general populace. It could differ depending on certain sociodemographic or cultural factors. This research examined women with ACS who also have coronary angiograms. This might not accurately represent the angiographic profile of women.

## Conclusions

This study reflects that coronary angiographic findings in premenopausal women revealed less severe lesions and single-vessel disease, and most commonly involved LAD, with low Friesinger scores compared to postmenopausal women. For further details, a comprehensive community-based study ought to be conducted.

## References

[1] Rosano GM, Panina G (1999). Oestrogens and the heart. Therapie.

[2] The Heart Truth (2023). The Heart Truth. https://www.nhlbi.nih.gov/health-topics/education-and-awareness/heart-truth.

[3] Yihua L, Yun J, Dongshen Z (2017). Coronary artery disease in premenopausal and postmenopausal women. Int Heart J.

[4] Barrett-Connor E (1997). Menopause: problems and interventions in the United States. Women’s Health and Menopause. Medical Science Symposia Series.

[5] Ahmed R, Das PR, Tushar AZ, Saha T (2021). Comparison of angiographic severity of coronary artery disease between premenopausal and postmenopausal women with acute coronary syndrome. Bangladesh Heart J.

[6] Soman B, Rahaman MA, Rajan R, Vijayaraghavan G (2016). Risk factor profile and disease pattern in premenopausal and postmenopausal Indian women presenting with acute coronary syndrome. J Clin Prev Cardiol.

[7] Nambirajan J, Kumar PP, Chakkravarthi D, Jegadeesh J, Senthil AN (2020). Coronary artery disease in premenopausal versus postmenopausal women - risk factors, clinical profile and angiographic characteristics. IOSR-JDMS.

[8] Nelson HD (2008). Menopause. Lancet.

[9] Tang H, Li Z, Fan Y (2022). Differences in culprit lesions between premenopausal and postmenopausal women with acute coronary syndrome: an optical coherence tomography study. Can J Cardiol.

[10] Friesinger GC, Page EE, Ross RS (1970). Prognostic significance of coronary arteriography. Trans Assoc Am Physicians.

[11] Ringqvist I, Fisher LD, Mock M (1983). Prognostic value of angiographic indices of coronary artery disease from the Coronary Artery Surgery Study (CASS). J Clin Invest.

[12] Siddika A, Malik FT, Kalimuddin M (2023). Severity of coronary artery diseases among pre- and postmenopausal women with acute coronary syndrome: a hospital-based study in Bangladesh. Cureus.

